# Inheritance of Vertebral Number in the Three-Spined Stickleback (*Gasterosteus aculeatus*)

**DOI:** 10.1371/journal.pone.0019579

**Published:** 2011-05-16

**Authors:** Jussi S. Alho, Tuomas Leinonen, Juha Merilä

**Affiliations:** Ecological Genetics Research Unit, Department of Biosciences, University of Helsinki, Helsinki, Finland; University of Cambridge, United Kingdom

## Abstract

Intraspecific variation in the number of vertebrae is taxonomically widespread, and both genetic and environmental factors are known to contribute to this variation. However, the relative importance of genetic versus environmental influences on variation in vertebral number has seldom been investigated with study designs that minimize bias due to non-additive genetic and maternal influences. We used a paternal half-sib design and animal model analysis to estimate heritability and causal components of variance in vertebral number in three-spined sticklebacks (*Gasterosteus aculeatus*). We found that both the number of vertebrae (*h^2^* = 0.36) and body size (*h^2^* = 0.42) were moderately heritable, whereas the influence of maternal effects was estimated to be negligible. While the number of vertebrae had a positive effect on body size, no evidence for a genetic correlation between body size and vertebral number was detected. However, there was a significant positive environmental correlation between these two traits. Our results support the generalization-in accordance with results from a review of heritability estimates for vertebral number in fish, reptiles and mammals-that the number of vertebrae appears to be moderately to highly heritable in a wide array of species. In the case of the three-spined stickleback, independent evolution of body size and number of vertebrae should be possible given the low genetic correlation between the two traits.

## Introduction

Jordan [1] observed that fish species living at higher latitudes tended to have more vertebrae than those living at lower latitudes. This formed the basis for what is today known as ‘Jordan's rule’ [2]. Although Jordan's rule has been confirmed in a number of interspecific (review in [2])-and sometimes also in intraspecific (e.g. [3]–[5])–studies, its underlying causes remain unclear [2]. As pointed out by MacDowall [2], there is also a lack of discrimination between environmental and inherited causes of variation in vertebral number. In fact, although studies in inheritance of vertebral number have been conducted in several species (see Discussion), many of these have used methods that do not allow additive genetic effects to be distinguished from maternal, early environmental and non-additive genetic effects.

The three-spined stickleback has become an important model organism in evolutionary biology and developmental research [6]–[8]. In particular, lateral plate variation has been studied extensively (reviewed in [9]), but less attention has been paid to body size and vertebral number variation. However, earlier studies have revealed that vertebral number can be under directional natural selection [10]–[11], and that they appear to have a heritable basis [12]. However, the available heritability estimates do not fully account for confounding environmental and maternal effects, and the relationship between number of vertebrae and body size has not been investigated.

The aim of this study was to investigate the relative roles of genetic, environmental and maternal effects on the number of vertebrae in three-spined sticklebacks, as well as to estimate heritability of this trait. To this end, we performed a large number of half-sib crosses on marine sticklebacks and subjected the data to ‘animal model’ analyses. In addition, we investigated the relationship between body size and the number of vertebrae, and tested whether the increase in vertebral number results in the increase of body size. Additionally, the genetic and environmental correlations between body size and number of vertebrae were estimated.

## Results

In all three cases-univariate analyses of vertebral number and body size, and multivariate analysis of both traits-only the models that included the additive effects received the strongest support (Table 1). Hence, the data do not lend support to the existence of any substantial maternal effects, either in the number of vertebrae or in body size.

**Table 1:** Model selection of genetic models.

Trait(s)	Model type	Random effects	DIC	ΔDIC
Body size	Univariate	**Additive genetic**	66.0	0.0
		Additive genetic+Maternal	96.8	30.8
		Maternal	124.3	58.3
		None	159.6	93.6
Number of vertebrae	Univariate	**Additive genetic**	561.7	0.0
		Additive genetic+Maternal	574.3	12.6
		Maternal	591.7	30.0
		None	616.8	55.1
Body size, number of vertebrae	Bivariate	**Additive genetic**	543.4	0.0
		Additive genetic+Maternal	646.5	103.1
		Maternal	701.1	157.7
		None	772.6	229.2

Model selection based on deviance information criterion (DIC). Most parsimonius model in bold.

The mean number of vertebrae was 31.74 (S.E. = 0.03, range = 29−34, n = 342), and there was no difference in the mean number of vertebrae between the sexes (difference [male-female] = 0.046, 95% HPDI: −0.070–0.167). There was significant additive genetic variation in vertebral number (Table 2), and the heritability of this trait equaled *h^2^* = 0.357 (Table 2).

**Table 2:** Heritability of vertebrae number and body size.

	Number of vertebrae		Body size	
Source	Var (95% HPDI)	*h^2^* (95% HPDI)	Var (95% HPDI)	*h^2^* (95% HPDI)
V_A_	0.135 (0.038–0.248)	0.357 (0.104–0.603)	0.043 (0.016–0.072)	0.445 (0.188–0.692)
V_R_	0.239 (0.148–0.325)		0.052 (0.031–0.076)	
V_P_	0.374		0.095	

Heritability (*h^2^*) and sources of variation in vertebrae number and body size in three-spined sticklebacks. V_A_ = additive genetic variance, V_R_ = residual variance, V_P_ = total phenotypic variance.

The mean body size was 40.62 mm (S.E: = 17.29, n = 338), but there was significant sexual size dimorphism, with females (mean body size = 41.45±0.24 [S.E.] mm, n = 167) being on average larger than males (mean body size = 39.74±0.22 mm; mean difference [male-female] = −0.176 mm, 95% HPDI: −0.231–−0.115). There was significant additive genetic variance also for body size (Table 2), with heritability equaling *h^2^* = 0.445 (Table 2). Fitting the number of vertebrae as a covariate into this analysis revealed that the body size increased as a function of number of vertebrae (*b* = 0.082, 95% HPDI: 0.027–0.134), but other effects in the model remained qualitatively unaffected.

Genetic correlation between number of vertebrae and body size was negative but not significant (*r_g_* = −0.324 [95% HPDI:−0.793–0.162]). However, environmental correlation between the traits was positive and significant (*r_e_* = 0.424 [0.116–0.760]). Since phenotypic correlation is a sum of genetic and environmental correlations [13], the weak positive association (*r_p_* = 0.104) observed above is largely driven by environmental effects.

Standard generalized mixed model analysis produced estimates of heritabilities (body size, *h^2^* = 0.51, 95% HPDI: 0.20–0.86; number of vertebrae, *h^2^* = 0.29, 95% HPDI: 0.05–0.58) that largely agreed with the animal model estimates. The slightly lower estimate for vertebral number in this alternative approach could be due to the fact that animal model estimates did not account for maternal (and common environment; see Methods) effects. Although non-significant, they accounted in the standard generalized mixed model analysis for 2.9% (95% HPDI: −11.8–17.4%) and 6.4% (95% HPDI: −9.7–18.3%) of the total phenotypic variance in body size and vertebral number, respectively. The estimate of genetic correlation in this alternative approach was *r_g_* = 0.071 (95% HPDI: −0.517–0.626), the correlation of maternal (or common environment) effects *r_m_* = −0.316 (95% HPDI: −0.782–0.195), and environmental (or residual) correlation *r_e_* = 0.211 (95% HPDI: 0.101–0.331). These estimates indicate that the non-significant negative genetic correlation observed with the animal model might in fact stem from maternal or common environment effects.

## Discussion

This study revealed that both body size and number of vertebrae in the three-spined stickleback are heritable, and that within population variation in body size is positively correlated to the number of vertebrae. However, this positive effect of vertebral number on size appears to stem from non-genetic influences, as the genetic correlation between the two traits was, depending on the analytical approach, either negative or weak, and in any case non-significant. Hence, the positive effect of vertebral number on body size appears to be due to environmental sources of variation, which influence both traits in a correlated fashion.

Although subject to much research over decades (e.g. [7], [14]), relatively few studies have investigated heritability of morphometric traits in three-spined sticklebacks ([12], [15]–[19]; see also [20]). Of these, only Hermida et al. [12] have looked at heritability of vertebral number. Comparison of our estimates with those of Hermida et al [12] is not straightforward. Our estimates from laboratory-reared fish were based on a paternal half-sib design, which is efficient in separating additive genetic effects from confounding environmental and non-additive effects [13], whereas those of Hermida et al. [12] were based on full-sib estimates known to be sensitive to inflation due to these effects [13]. Hence, the tendency for higher heritabilities (*h^2^* = 0.44−0.55) from [12] as compared to this study (*h^2^*≈0.36) could owe to methodological differences. This is also suggested by the fact that regression estimates of heritabilites in the Hermida et al. [12] study were much lower (and non-significant) than the full-sib estimates (Table 3).

**Table 3:** Heritability of number of vertebrae in different species.

Taxon	Species	n_F_/n_I_	*h^2^*±S.E.	Type	Reference
Fish	Belly shark, *Etmopterus spinax*	23/224	0.59±0.21	FS	[35]
	Medaka, *Oryzia latipes*	134/?	0.32±0.07	MM*	[30]
		?	0.90	FS	[27] ††
	Eelpout, *Zoarces viviparious*	?	0.81	OM	[36]
	Japanese flounder, *Paralichthys olivaceus* (hatchery)	31/63	0.64±0.07	AM	[37]
	(wild)	33/50	0.52±0.12	AM	[37]
	Coho salmon, *Oncorhynchus kisutch* (1994)	6/262	0.64	FS	[38]
	(1995)	13/455	0.69	FS	[38]
	Masu salmon, *Oncorhynchus masu*	10/500	0.65±0.20	MM	[39]
	Rainbow trout, *Oncorhynchus mykiss*	?	0.66	RH	[40] ††
	Carp, *Cyprinus carpio*	?	0.86	RH	[41] ††
		?	0.65	?	[42] ††
		?	0.90	?	[42] ††
	Guppy, *Poecilia reticulata*	14/1412	0.38	LC	[43]
	Brown trout, *Salmo trutta*	?	0.90	MM	[44] ††
	Threespine stickleback, *Gasterosteus aculeatus*	33/?	0.51±0.16	FS**	[12]
		33/?	0.24±0.22	MM**	[12]
		48/342	0.36	AM	This study
	Nine-spined stickleback, *Pungitius pungitius*	10/81	1.22±0.40†	MM	[45]
Reptiles	Garter Snake, *Thamnophis elegans* (costal)	94/780	0.65±0.14	FS	[24]
	(inland)	159/1459	0.79±0.11	FS	[24]
	Adder, *Vipera berus*	29/213	0.39±0.14	FS	[46]
	Japanese Mamushi snake, *Gloydius blomhoffii*	10/≈24	0.71±0.30	FS	[26]
Mammals	Domestic pig, *Sus scrofa*	4784/?	0.74	MM	[47]
	Domestic pig, *Sus scrofa*	+120/4258	0.62±0.06	AM	[25]

Heritability (*h^2^*) of number of vertebrae in different species. Type refers to heritability estimation method (FS = full-sib heritability, MM = midoffspring-midparent regression, AM = animal model, RH = realized heritability, LC = line-cross). n_F_ = number of families, n_I_ = number of individuals. ‘?’ denotes missing information.

*Pooling 13 populations.

**average of separate estimates for caudal and abdominal estimates.

† Calculated from data in Table 1 in [45].

†† Estimates taken from Table 19 in [36].

Depending on study design, maternal and non-additive genetic effects can confound estimates of additive genetic variance and heritability. The breeding design we have employed in this study should be robust in respect to both of these factors. In our design, most non-additive effects should end up in residual variance, whereas maternal effects could either increase or decrease additive genetic variance. We did take maternal effects into account in the model selection-fitting models that included them–but since no evidence for them was found, maternal effect terms were excluded from final parameter estimation. Also, the standard generalized mixed model analyses–in which the dam effects were explicitly fitted into the model–suggested that maternal effect influences were small (2–7% of total variance explained) relative to additive genetic effects. However, these analyses also suggest that the small discrepancy between animal model (*h^2^* = 0.36) and standard generalized mixed model (*h^2^* = 0.29) estimates of heritability for vertebral number could be due to unaccounted maternal effects (*m* = 0.06) in the animal model estimate.

Although heritabilities of vertebrae counts have been estimated in a number of earlier studies, this body of work–conducted in various reptilian, fish and mammalian species–has apparently never been reviewed. Although the aim of this study was not to conduct any comprehensive review of this topic, the compilation of published heritability estimates for vertebral counts in Table 3 provides some insights. It is noteworthy that the heritability estimates for number of vertebrae are generally very high: the median estimate in Table 3 is 0.650, which is much higher than that for morphological traits in general (0.461, S.E. = 0.004; [21]). However, as many of the estimates are based on full-sib analyses, it is possible that some may be inflated by maternal and common environmental effects. Nevertheless, several of the estimates obtained using parent-offspring regressions are high as well, suggesting that heritabilities of number of vertebrae are generally very high. This is interesting in light of the inverse correlation between trait heritability and its importance to fitness [22]. In other words, the high heritability of vertebral number suggests that it is unlikely to be a trait under strong and consistent directional selection.

According to Jordan's Rule [1], [2], the number of vertebrae is expected to increase towards higher latitudes-a pattern which has been observed also in the case of mean body size in several species of fishes [23]. A positive correlation between body size and vertebral number would be expected if the number of vertebrae allows individuals to grow large. Indeed, such a correlation has been earlier documented in some studies focusing on within population variation (e.g. [24]–[26]). We also found a positive association between number of vertebrae and body size in the three-spined stickleback. However, this effect was rather weak, and interestingly, apparently non-genetic, as the genetic correlation between the number of vertebrae and body size was negative and non-significant. Instead, the positive correlation appeared to be due to positive environmental covariance between body size and vertebral number. Hence, some as yet unidentified environmental factor(s) exerting a positive influence on the number of vertebrae also appear to influence individual growth.

Finally, we note that the vertebral counts in fish appear to be strongly influenced by temperature experienced during their early development (e.g. [27]–[28]). Consequently, the amount of additive genetic and environmental variance–and thereby heritability–in vertebral counts may differ depending on the rearing conditions (e.g. [29]). Given that natural environments are likely to be more heterogeneous than standardized laboratory environments, it is likely that the environmental component of variance is higher in the wild than in the laboratory. If so, this could explain why the heritabilities in Table 3 are so high. That heritability estimates of vertebral number can be influenced by environmental conditions has been demonstrated e.g. by Yamahira et al. [30]. Studying medaka, they observed that the heritability vertebral number declined as function of increasing rearing temperature. It has also been shown for three-spined sticklebacks that the correlation between the vertebral number of mother and offspring can change from positive to negative depending on temperature [31].

In conclusion, the results of this study confirm that the number of vertebrae in three-spined sticklebacks is heritable, and that variation in vertebral number is positively associated with variation in body size in the same population. However, the positive correlation between body size and number of vertebrae appears to be non-genetic, suggesting that selection on either body size or number of vertebrae would not necessarily result in correlated response in either trait. Whether the positive correlation between body size and vertebral number occurs also among different three-spined stickleback populations remains to be investigated.

### Ethics statement

The breeding experiment in this study was conducted in accordance with Finnish laws and guidelines with permission from the University of Helsinki Animal Experimentation Board (permission # STH379A/HY121-06).

## Materials and Methods

The parents for the fish used in this study were collected in June 2006 from the Baltic Sea near Helsinki (Vuosaari, Helsinki; 60°10′N, 25°00′E) for the purpose of a large genetic experiment [18]–[19]. In short, a paternal half-sib design was employed using 42 males, each mated with two independent females resulting in 84 full-sib families. Fertilizations were done as explained in Leinonen et al. [18]–[19] and the eggs from these crosses were raised in 17°C until hatching. After hatching, the fry from each family were divided into two replicates, and 15 fish/family/block from each of the 84 families were raised to an age of 190 days, after which the fish were killed with an overdose of MS-222. 4–5 fish from each family were used for this experiment (vertebra counts). The killed fish were fixed in 10% formalin for approximately one month, after which they were stored in 70% ethanol. Leinonen et al. [18]–[19] give more details about rearing conditions and feeding regime of the experimental fish.

The vertebrae were counted under dissection microscope after the whole vertebral column from one side was exposed with the aid of a scalpel. To determine the repeatability of vertebral number counts, 20 individuals were counted twice and blind with respect to the first count. Repeatability (R) was estimated following [32] and found to be R = 1 (F_19,20_>3630.99, P<0.001). Hence, counting (measurement) error was zero. All the counts were made by the same person. No distinction was made between abdominal and caudal vertebrae, but all vertebrae were counted. Fused vertebrae were counted as two.

Sex of the individuals was identified by amplifying a part of 3′UTR of the IDH gene as explained in [18]. This method is based on the logic that the primers amplify two fragments (∼280 bp and 300 bp) in males, but only one fragment (300 bp) in females [18]. From each fish, we also measured standard length (from anterior tip of the upper lip to the end of the caudal peduncle to get an estimate of body size. This measure is strongly correlated with multivariate measures of body size (PC1 and centroid size) calculated from this data based on 17 landmark measurements (cf. [19]).

Contributions of genetic and environmental effects on vertebral number and body size were estimated for individuals measured for both traits (on average 3.98 [median = 4] fish from each of the 84 families) using animal model analyses as implemented in MCMCglmm package [33]. To this end, we fitted the univariate model:
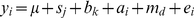
(1)Where *y_i_* is the observed trait value, *j* the sex, *k* the block and *d* the dam of individual *i*, *μ* the intercept, *s_j_* fixed effect of sex, *b_k_* fixed effect of block, *a_i_* the random additive genetic effect, *m_d_* the random effect of maternal identity estimating maternal effects influences, and *e_i_* the residual or environmental effect. Apart from fitting this model, in the case of body size we also fitted a model in which vertebral number was added as a covariate. The rationale behind this was to see whether any of variance in the body size could be explained by variation in vertebral number. Also a multivariate model was fitted to estimate genetic and environmental correlations between body size and vertebral number. Normal distribution was assumed for all random effects in all models, and commonly used prior specification for variance components (Inverse-Wishart, V = 1, nu = 0.002) was used for univariate and (V = diag(2) * 0.02, nu = 3) for multivariate models. For each model, one chain with 600,000 iterations–first 100,000 of which discarded as burnin–was run. The chain was thinned by 100, resulting in 5,000 samples from posterior distribution.

In all three cases–univariate analyses of vertebral number and body size, and multivariate analysis of both traits–we used model selection procedure based on deviance information criterion (DIC) to choose the most parsimonious model: the smaller the DIC-value, the more parsimonious the model. The set of candidate models in each case included models with both additive and maternal effects included and excluded. In order for a more complex model to be favored over a more parsimonious one, the difference between DIC-values of the two models (ΔDIC) should not exceed 10 [34]. The reported estimates of heritability, variance components, sex differences, and the effect of vertebral number on body size are based on the univariate analyses, whereas the estimates of genetic and environmental correlations are from the multivariate analysis. Unless otherwise noted, the estimates are reported as posterior means and 95% highest posterior density intervals (95% HPDIs).

For comparative purposes we also report heritability estimates and genetic, maternal and environmental correlations between body size and vertebral number as obtained from standard generalized mixed model analysis implemented in the MCMCglmm package. Sire and dam (nested within sire) effects were treated as random effects, and sex and block as fixed effects. A single multivariate model was fitted, and again commonly used prior specification for variance components (V = diag(2) * 0.02, nu = 3) was used. One chain with 600,000 iterations–first 100,000 of which discarded as burnin–was run. The chain was thinned by 100, resulting in 5,000 samples from posterior distribution. In this analysis the variance component attributable to sire effect is ¼ V_A_ ( = additive genetic variance) and the variance component attributable to dam is the sum of ¼ V_A_, ¼ V_D_ ( = dominance variance) and V_M_ ( = maternal effect variance, including common environment effects [13]). If we assume that V_D_ = 0, then subtracting the sire component from the dam component gives us an estimate of maternal (including common environment) effect component. Genetic correlation was estimated following equation 19.3 in Falconer and Mackay [13].
